# Insulin-like Growth Factor-Binding Protein-1 (IGFBP-1) as a Biomarker of Cardiovascular Disease

**DOI:** 10.3390/biom14111475

**Published:** 2024-11-20

**Authors:** Moira S. Lewitt, Gary W. Boyd

**Affiliations:** 1 School of Health and Life Sciences, University of the West of Scotland, Paisley PA1 2BE, UK; 2 School of Health and Life Sciences, University of the West of Scotland, Hamilton G72 0LH, UK; gary.boyd@uws.ac.uk

**Keywords:** insulin-like growth factor/IGF, IGF-binding protein-1/IGFBP-1, insulin resistance, cardiometabolic risk

## Abstract

Insulin-like growth factor-binding protein-1 (IGFBP-1) contributes to the regulation of IGFs for metabolism and growth and has IGF-independent actions. IGFBP-1 in the circulation is derived from the liver, where it is inhibited by insulin and stimulated by multiple factors, including proinflammatory cytokines. IGFBP-1 levels are influenced by sex and age, which also determine cardiometabolic risk and patterns of disease presentation. While lower circulating IGFBP-1 concentrations are associated with an unfavorable cardiometabolic risk profile, higher IGFBP-1 predicts worse cardiovascular disease outcomes. This review explores these associations and the possible roles of IGFBP-1 in the pathophysiology of atherosclerosis. We recommend the evaluation of dynamic approaches, such as simultaneous measurements of fasting IGFBP-1 and proinsulin level in response to an oral glucose challenge, as well as multi-marker approaches incorporating markers of inflammation.

## 1. Introduction

The insulin-like growth factors, IGF-I and IGF-II, are part of a complex system that includes IGF receptors and IGF-binding proteins (IGFBPs), that has an important role in coupling growth, metabolism and reproduction [1]. The IGFBPs are proteins that bind IGFs with high affinity and determine their availability to receptors on the cell surface, and, therefore, activity of the IGFs. Studies of IGF-I as a marker of cardiovascular disease have yielded inconsistent results [2,3], however, one of the IGFBPs, IGFBP-1, has emerged as a more promising biomarker and is, therefore, the focus of this review. The aim in searching for cardiovascular disease biomarkers is broadly twofold: (i) to identify at-risk individuals in order to personalize approaches to disease prevention and management, and (ii) to understand the pathophysiology in order to identify novel therapeutic targets [4]. These include biomarkers of insulin resistance and chronic inflammation, including adipokines [4]. This review will focus on the published evidence for IGFBP-1 as a potential biomarker. The physiological roles of IGFBP-1 will first be described with an emphasis on why it is regarded as a marker of hepatic insulin action. The association between circulating IGFBP-1 concentrations and cardiovascular disease risk and outcomes will then be reviewed, followed by an exploration of possible roles in the pathophysiology of atherosclerosis. Finally, consideration will be given as to how IGFBP-1 might be explored as a biomarker for use in personalized care, along with recommendations for future research.

## 2. IGFBP-1 in Physiology

### 2.1. IGFBP-1 Protein Structure and Function

IGFBP-1 is a ~30 kDa member of the family of proteins. The six human IGFBPs have conserved disulfide-linked cysteines in *N*- and *C*-terminal domains that together determine high-affinity IGF binding, and are linked by a more variable central domain that confers functional differences between IGFBPs [5,6,7]. In the case of IGFBP-1, the central domain has serine phosphor-acceptor sites. When phosphorylated, IGFBP-1 has a high affinity for IGFs [8] and inhibits IGF availability to type 1 IGF receptors (IGF1R), insulin receptors and their hybrids [5]. The *C*-terminal domain of IGFBP-1 has an Arg-Gly-Asp region that binds to the α_5_β_1_ integrin. IGFBP-1 has been shown to stimulate the migration of Chinese hamster ovary cells [9] and human trophoblasts [10] by this IGF-independent mechanism.

In the circulation, IGFs are mostly associated with IGFBP-3 and ALS in growth hormone (GH)-dependent ternary complexes of ~140 kDa [11]. These high molecular mass forms cannot cross the endothelial barrier. In contrast, IGFs unbound (‘free IGFs’) or in binary complexes with IGFBPs leave the circulation with half-lives of minutes to hours [12]. In adult humans, IGFBP-1 is mainly expressed in the liver, and variations in circulating IGFBP-1 concentrations are a result of changes in IGFBP-1 synthesis by hepatocytes. IGFBP-1 is secreted in a phosphorylated state, with Ser^101^ as the major site of phosphorylation [8]. Recent studies suggest that nutrient deprivation increases the phosphorylation state of IGFBP-1. In HepG2 cells, there is a hyperphosphorylation response to leucine deprivation via activation of protein kinase Cα [13] and to glucose deprivation by the AMPK/mTORC1 pathway [14].

IGFBP-1 in the circulation is mainly phosphorylated and when it crosses the endothelium, it is available to inhibit IGF action in peripheral tissues. Phosphorylated IGFBP-1 is more potent than non-phosphorylated IGFBP-1 at inhibiting the mitogenic effect of IGF-I on 3T3-L1 preadipocytes [15]. After dephosphorylation in peripheral tissues, the affinity of IGFBP-1 for IGF-I is reduced several-fold [8]. Depending on the cellular milieu, non-phosphorylated IGFBP-1 can potentiate the effect of IGF-I, e.g., on smooth muscle DNA synthesis [16]. An IGFBP-1-specific protease activity has been described that cleaves phosphorylated IGFBP-1 at Ile^130^-Ser^131^ to produce fragments with low affinity for IGFs [17]. Although the protease activity is inhibited by inhibitors in serum, these fragments may be present in the circulation, suggesting cleavage at the tissue level [18].

### 2.2. IGFBP-1 Regulation

Early studies indicated that circulating IGFBP-1 is dynamically regulated with a marked diurnal variation related to food intake [19] and it was proposed that IGFBP-1 might have a glucose counterregulatory role, inhibiting the availability of IGFs for insulin-like actions in the fasting state [20]. Studies using HepG2 human or H4-II-E rat hepatoma cells have consistently demonstrated that insulin inhibits IGFBP-1 transcription [21]. In human studies in vivo, insulin concentrations within the physiological range suppress serum IGFBP-1 [22,23]. IGFBP-1 is also regulated by a range of stimulators, including hormones involved in glucose counter-regulation. In HepG2 cells, glucagon and glucagon-like peptide-1 have post-transcriptional stimulatory effects on IGFBP-1 release [24]. In vivo, infusions of glucagon [25] and epinephrine or norepinephrine [26] stimulate IGFBP-1. Cortisol has also been shown to stimulate IGFBP-1 in vivo under conditions of hypoinsulinemia [27].

The proinflammatory cytokines, interleukin (IL)-1β, IL-6 and tumor necrosis factor (TNF)-α each stimulate IGFBP-1 transcription [28] independently of the small stimulatory effect of reactive oxygen species, including H_2_O_2_ and nitric oxide donors [28]. Proinflammatory cytokines reduce hepatic insulin sensitivity [29] and, therefore, potentially also influence IGFBP-1 production via that mechanism. The very high levels of IGFBP-1 seen in critically ill patients [30] are likely due to a combination of all these factors.

**Figure 1:**
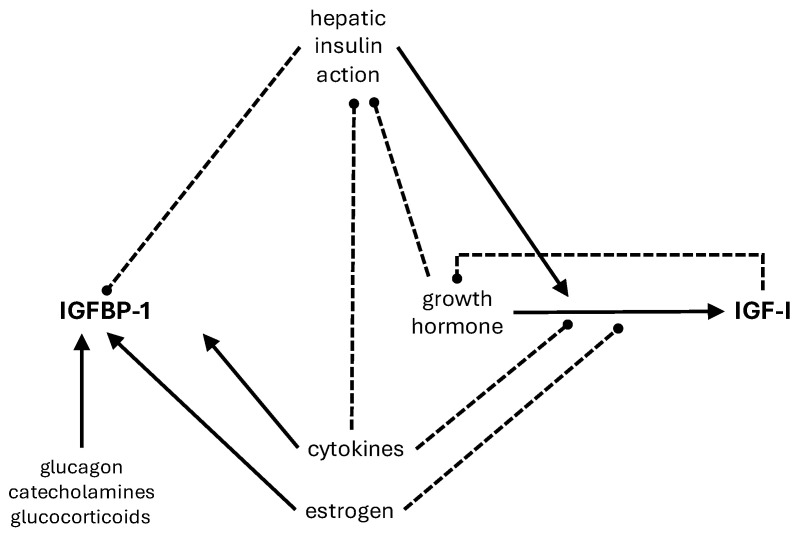
Regulation of hepatic IGFBP-1 and IGF-I Stimulatory effects are indicated by the solid lines and inhibitory effects by the broken lines.

Many of the regulators of IGFBP-1 also regulate IGF-I production and actions (see Figure 1). Insulin, which inhibits IGFBP-1, promotes the stimulatory effect of GH on IGF-I synthesis [31]. Estrogen stimulates IGFBP-1 but reduces hepatic sensitivity to GH [32], and, therefore, the GH effect on IGF-I. Pro-inflammatory cytokines inhibit IGF production and also induce IGF resistance [33].

An understanding of the factors regulating IGFBP-1 is important in interpreting levels in studies of cardiovascular risk. The timing of a sample in relation to food intake is important, and, therefore, most studies report fasting concentrations. The sex and age of an individual are important. IGFBP-1 is higher in females compared to males [34,35,36]. This is attributed to the action of estrogen. In monozygotic twins discordant for oral contraceptive use, IGFBP-1 increased markedly in response to oral contraceptives [37]. And, in postmenopausal women, oral estrogens increase IGFBP-1 concentrations [38,39]. In older adults, IGFBP-1 increases positively with age [40,41].

### 2.3. IGFBP-1 as a Marker of Hepatic Insulin Action

As detailed above, insulin transcriptionally inhibits IGFBP-1 production by hepatocytes and determines the inverse relationship between circulating concentrations of insulin and IGFBP-1. In obesity, with peripheral insulin resistance and, therefore, decreased responsiveness to insulin-mediated glucose disposal or adipose tissue lipolysis, insulin concentrations increase and IGFBP-1 is suppressed. In population studies, this is represented by movement down along the regression line between IGFBP-1 and insulin [23,42,43]. IGFBP-1 concentrations correlate inversely with BMI and measures of adiposity, e.g., waist circumference in females and males [35,36,44]. Central obesity, determined by ethnicity-specific waist circumference measurement, is a core criterion in the International Diabetes Federation’s definition of metabolic syndrome [45]. The other criteria, comprising two of the following, raised triglycerides, reduced HDL-cholesterol, raised blood pressure, and raised fasting glucose, are accepted cardiovascular risk factors [45]. Individuals with metabolic syndrome are at high risk for glucose dysregulation. The presence of metabolic syndrome is a stronger predictor of type 2 diabetes (T2D) than coronary heart disease; nevertheless, it serves as a simple clinical tool for identifying those at high risk of cardiovascular disease or T2D [46].

IGFBP-1 is determined by portal insulin concentrations and hepatic insulin sensitivity. In individuals without diabetes, there is a portal-peripheral insulin gradient [47] indicating clearance of insulin by the liver [48]. In the absence of endogenous insulin secretion, in type 1 diabetes (T1D), circulating IGFBP-1 concentrations are disproportionately increased in relation to whole-body insulin sensitivity, measured by a euglycemic hyperinsulinemic clamp [49]. However, when portal insulin concentrations are estimated, IGFBP-1 concentrations are appropriate [49]. It has been observed that proinsulin, which has lower hepatic extraction, has a stronger relationship with IGFBP-1 than insulin [44,50,51].

In T2D, there is an inverse relationship between IGFBP-1 and insulin however, a parallel upward shift in the regression line is observed, compared to pre-diabetes [36,44]. This is consistent with the emergence of hepatic insulin resistance, including reduced hepatic insulin extraction. Theoretically, this may also be due to a chronic low-grade inflammation with increased proinflammatory cytokines that stimulate IGFBP-1. Hepatic fat accumulation is closely linked to the development of hepatic insulin resistance [52] and may be a key component of metabolic syndrome [45]. Fasting IGFBP-1 concentrations are inversely related to fat liver content and have been shown to correlate with hepatic insulin sensitivity, better than fasting insulin levels [53].

## 3. IGFBP-1 in Cardiovascular Diseases

Cross-sectional and prospective studies demonstrate associations between IGFBP-1 and cardiovascular diseases. As detailed above, IGFBP-1 concentrations are determined in part by sex (levels (higher in females) and age (higher in older adults). These factors also have an impact on cardiovascular risk [54,55,56]. Therefore, details of the study population (participant number (n), age in years (y) and percentage female (%F)) will be included in the following discussions.

### 3.1. IGFBP-1 as a Predictor of Cardiovascular Disease

Some prospective longitudinal cohort studies report that higher fasting serum IGFBP-1 levels predict higher cardiovascular disease mortality. In the Framingham population study (*n* = 3523, mean 62 y, 53% F) over nearly 30 years, higher IGFBP-1, IGFBP-2 and IGF-I predicted cardiovascular disease mortality [57]. A male cohort (*n* = 622, 65–84 y) showed that higher IGFBP-1, after adjustment for age, was associated with an increased risk of cardiovascular mortality over a five-year period [58]. In another study in males (*n* = 3983, ≥70 y), with a mean follow-up of five years, higher IGFBP-1 and lower IGFBP-3 predicted all-cause and cardiovascular mortality [59].

Other prospective studies report lower fasting IGFBP-1 or no association with future cardiovascular risk. In healthy older adults (*n* = 1185, 51–98 y, 47% F), lower fasting IGFBP-1 and lower total IGF-I were independently and jointly associated with ischemic heart disease mortality, but not all-cause mortality during a 9- to 13-year follow-up [60]. In males (*n* = 187, <60 y) with previous myocardial infarction, low total and not easily dissociable IGF-I, IGFBP-3, IGFBP-5 and ALS were associated with angiographically assessed coronary heart disease [61]. In this study, there was no association with IGFBP-1 levels. The relationship between lower total IGF-I and increased risk of ischaemic heart disease was supported by a nested case-control study stratified by age and sex, during a 15-year follow-up [62]. In a large case-cohort study, there was a U-shaped relationship between IGF-I levels and mortality from CVD, with those with the lowest and highest having the greatest risk [63]. This relationship was attenuated when those with liver dysfunction were excluded from the analysis.

Cross-sectional studies suggest that lower IGFBP-1 is associated with a cardiovascular risk profile. In a cross-sectional study of older adults (*n* = 218, 55–80 y, 53% F), lower fasting IGFBP-1 was associated with a less favorable cardiovascular risk profile, independently of fasting insulin [64]. In this study, higher easily dissociable IGF-I was associated with decreased presence of atherosclerotic plaques and coronary artery disease, while IGFBP-1 was not. Individuals with a history of ischemic heart disease (*n* = 75, mean 50 y) have lower IGFBP-1, particularly lesser phosphorylated forms, compared to controls [65]. In this study, phosphorylated IGFBP-1 correlated inversely with fasting insulin, and homeostatic model assessment-insulin resistance (HOMA-IR).

### 3.2. Acute Coronary Syndrome

Acute coronary syndrome refers to a range of clinical presentations including acute myocardial infarction and unstable angina. On admission, serum IGFBP-1 may be low or high, compared to controls. In a study of individuals with acute coronary syndrome (*n* = 112, 26–83 y, 41% F), IGFBP-1 was positively associated with patient age and, in age-comparable patients, IGFBP-1 was increased in critical coronary artery disease [66]. In another study of acute myocardial infarction (*n* = 34, mean 34 y, 17% F), lower IGFBP-1 and higher easily dissociable IGF-I and IGFBP-3 levels were observed, compared to age- and sex-matched controls [67]. In patients presenting with unstable angina, those with critical coronary artery disease (*n* = 67, mean 62 y, 25% F) had higher IGFBP-1 than those with noncritical disease (*n* = 45, mean 60 y, 64% F) [68]. In that study, while the correlation with age was preserved, there was no correlation between IGFBP-1 and gender.

In younger males surviving a first myocardial infarction (*n* = 92, <45 y), IGF-I and not IGFBP-1 was independently associated with coronary artery disease progression over a five-year period [69]. Other prospective cohort studies suggest higher IGFBP-1 concentrations at presentation are associated with poorer long-term outcomes. Higher IGFBP-1 in acute myocardial infarction was associated with all-cause mortality but not with cardiovascular events over a median follow-up of 12 years (*n* = 180, median 64 y, 31% F) [70]. In a study of survivors of a first acute myocardial infarction (*n* = 853, mean 60 y, 34% F), higher IGFBP-1 predicted heart failure as well as mortality over an eight-year follow-up [71]. In that study, higher IGFBP-1 also predicted heart failure in the control group (*n* = 1106, mean 61 y, 36% F). In T2D patients surviving acute myocardial infarction (*n* = 1253, mean 70 y, 33% F), higher levels of IGFBP-1 at admission predicted cardiovascular morbidity and mortality during a median follow-up of two years [72].

### 3.3. Heart Failure

As described above, higher IGFBP-1 has been identified as a long-term predictor of heart failure (HF) [71]. In older individuals (*n* = 566, mean 74 y, 58% F), higher IGFBP-1 predicted incident HF over a six-year follow-up [73]. In a study of patients with worsening signs and symptoms of HF, IGFBP-1 was higher in ischemic HF (*n* = 715, 63–78 y, 19% F) relative to non-ischemic HF (*n* = 445, 54–73 y, 27% F) [74]. In cardiomyopathy, IGFBP-1 concentrations were higher and total IGF-I lower in those with a history of HF (*n* = 18, mean 65 y, 33% F) compared to those without (*n* = 106, mean ~58 y, 26% F) [75]. In that study, IGFBP-1 increased while IGF-I further decreased in the heart failure group over a mean 53-month follow-up period.

Higher IGFBP-1 also predicts adverse outcomes in those with HF. In outpatients with chronic HF, higher IGFBP-1, along with IGFBP-2, predicts adverse clinical outcomes (*n* = 263, mean 67 y, 28% F) [76]. In that study, measures of each biomarker were repeated at 3-month intervals for 2.2 years, and the slopes, representing an increase in IGFBP-1 and IGFBP-2, also predicted adverse clinical outcomes. In HF with reduced ejection fraction (*n* = 537, 58–76 y, 26% F), IGFBP-1 was part of an optimal set of biomarkers identified as useful in the prediction of adverse clinical events over a median of 2.2 years [77]. In a study of proteomics in heart failure (*n* = 1134, mean 70 y, 32% F), IGFBP-1 demonstrated the largest treatment effect, with a fall of more than 70% in response to the sodium–glucose cotransporter 2 inhibitor empagliflozin [78].

Atrial fibrillation often coexists in patients with heart failure. In a cohort study of individuals with HF (*n* = 3378, mean 62, 54% F), higher IGFBP-1 and lower IGF-I concentrations were associated with a higher risk of incident atrial fibrillation over a mean follow-up of 12 years [79]. In another study of HF patients, IGFBP-1 was higher in those with atrial fibrillation (*n* = 648, mean 72 y, 25% F) compared to patients in sinus rhythm (*n* = 972, mean 65 y, 31% F) [80].

In heart failure with preserved ejection fraction (HFpEF), which accounts for more than half the cases of HF, coronary microvascular inflammation has an important role [81,82]. In network analysis of biomarker profiles (*n* = 1544, mean ~74 y, 32% F), IGFBP-1 did not emerge as a unique identifier of HFpEF compared to HF with reduced ejection fraction (HFrEF) [83]. Although IGFBP-1 levels were higher in both groups compared to controls, there was no difference between those with HFpEF (*n* = 79, mean 73 y, 52% F) and HFrEF (*n* = 85, mean 64 y, 16% F) [84]. In this study, total IGF-I predicted outcome in HFrEF. In a separate study, IGF-I was identified as one of the anabolic hormone deficiencies that are prevalent in HFpEF (*n* = 84, 59–98 y, 57% F) [85].

In a study of HFpEF (*n* = 96, mean 74 y, 59% F), higher IGFBP-1 is amongst a set of proteins, also including IGF-I, IGF-II and IGFBP-2, that together are associated with the presence of pulmonary hypertension and five-year mortality [86]. In that study, IGFBP-1 was also independently associated with all-cause mortality. While the prevalence of coronary microvascular dysfunction among females and males is similar, it has been suggested that the underlying pathophysiological mechanisms might differ by sex [87]. In a study of patients with HFpEF (182, mean 74 y, 57% F), IGFBP-1 was a strong protein correlate of coronary microvascular dysfunction in females [88].

### 3.4. Cerebrovascular Disease

Although it has long been recognized that the IGF system has a role in the rescue of neurons following hypoxic-ischemic injury [89], studies of IGFBP-1 as a marker of cerebrovascular disease are few. In a cross-sectional study in healthy males (*n* = 96, mean 50 y), higher fasting serum IGFBP-1 and lower easily dissociable IGF-I were each associated with intima-media thickness (IMT) and accounted for just 6% and 5% of the variation in IMT, respectively [90]. In this study, the inverse relationship between IGFBP-1 and insulin was preserved. In older independently living males (*n* = 403, 73–94 y), easily dissociable IGF-I was also inversely related to IMT, and there was no correlation with IGFBP-1 concentrations [91].

In the acute phase of ischaemic stroke (*n* = 470, mean 57 y, 36% F), higher serum IGFBP -1 is associated with a risk of poor functional outcome and of mortality over a seven-year follow-up [92]. Population studies show lower IGF-I [93,94] and lower IGFBP-3 [94] are associated with increased risk of morbidity and mortality from ischaemic stroke.

A single-nucleotide polymorphism in the IGFBP-1 gene is associated with hemorrhagic stroke (mean age 64 years, 39% female) [95]. Gene variants in *Igf1*, *Igf1r* and *GHRH* are associated with protection against the development of ischaemic stroke [95,96,97]. IGFBP-1 protein has been found to be expressed in human carotid plaques and may be associated with plaque inflammation [98].

### 3.5. Peripheral Vascular Disease

Studies of IGFBP-1 in peripheral vascular disease are notably lacking. IGFBP-1 is independently associated with increased aortic diameter in men (*n* = 3981, 70–89 y) [99].

Increased amounts of IGFBP-1 protein, along with increased IGFBP-3 and reduced IGF-I, have been found in aortic aneurysm tissues, including the mural thrombus [100]. Another study identified IGFBP-1 protein in the luminal part of the thrombus and levels correlated with the size of abdominal aortic aneurysm [101].

In T1D with peripheral vascular complications, hyperbaric oxygen therapy is associated with a fall in insulin, an increase in IGFBP-1 and an improved lipid profile [102].

## 4. Relationship Between IGFBP-1 and Other Cardiometabolic Markers

Insulin resistance is a key feature of the metabolic syndrome and is causally related to cardiovascular disease [103]. Studies described in the preceding section showed that circulating IGFBP-1 concentrations were inversely associated with markers of cardiovascular disease. Cross-sectional studies show that low IGFBP-1 concentrations are associated with adverse cardiovascular risk profiles, correlating inversely with insulin and measures of adiposity and positively with HDL-cholesterol [64,65,104].

In a cross-sectional study in children aged 11–15 years (42% female), low IGFBP-1 was an independent marker of insulin resistance (determined by fasting insulin and response to intravenous glucose) and was better than elevated triglycerides and decreased HDL and adiponectin levels [105]. In obese adolescents (mean age 14 years, 66% Tanner stage 4 or 5, 56% female), IGFBP-1 is strongly associated with insulin sensitivity measures including fasting insulin, the HOMA-IR and insulin sensitivity index (WBISI and S_I_) [106]. In both studies, there was a positive correlation between IGFBP-1 and adiponectin and HDL concentrations and an inverse correlation with triglyceride levels and anthropometric measures, including waist circumference. The relationship between IGFBP-1 and adiponectin remained after controlling for the influence of insulin sensitivity [106]. These studies suggested that low IGFBP-1 might be a better marker of cardiometabolic risk than traditional measures. One study suggests that IGFBP-2 might also be a promising marker of insulin resistance in children regardless of pubertal stage [107].

In young people with T2D (*n* = 699, 10–17 y, 65% F), there was an inverse relationship between IGFBP-1 and BMI [108]. In this cohort, higher BMI was associated with higher IL-6 levels, along with TNF receptors 1 and 2 and the acute phase reactants, high sensitivity C-reactive protein, plasminogen activator inhibitor 1 and fibrinogen. In young adults (*n* = 150, 18–35 y 22% F), fasting serum IGFBP-1 together with red blood cell count, alanine aminotransferase activity, serum C-peptide, sex hormone binding globulin and adiponectin correlated with insulin sensitivity determined by hyperinsulinemic-euglycemic clamp [109]. This model, based on a single fasting blood sample, reflected the change in insulin sensitivity with weight loss in individuals with prediabetes and diabetes [109]. In older males (*n* = 331, 70–89 y), low fasting IGFBP-1 correlated inversely with fasting insulin and insulin two hours after an oral glucose challenge, as well as body mass index [110].

Low IGFBP-1, high CRP and low IGF-I are independently associated with the presence of WHO-defined metabolic syndrome and insulin resistance in different ethnic groups (*n* = 440, mean 51 y, ~50% F) [111]. In a population cohort (*n* = 839, 40–65 y, 58% F), low IGFBP-1 (below the median) and high CRP (in the highest tertile) were together associated with a dramatic increase in the risk of metabolic syndrome (odds ratio 14) [112].

Low IGFBP-1 is a predictor of prediabetes and type 2 diabetes [36,44,113]. In a nested case-control study, when adjusted for age and BMI, IGFBP-2 and adiponectin levels were stronger independent markers in females, while in men, IGFBP-1 was the strongest marker [113]. In adults with established type 2 diabetes, lower IGFBP-1 remains associated with cardiovascular risk factors including fasting insulin, LDL cholesterol and BMI [114]. In a prospective study (*n* = 922, ≥65 y, 65% F), although low IGFBP-1 was associated with increased prevalence of glucose intolerance, low IGF-I and IGFBP-3 levels, and not IGFBP-1, were associated with increased IL-6 and CRP [115].

## 5. Pathogenesis of Atherosclerosis

**Figure 2:**
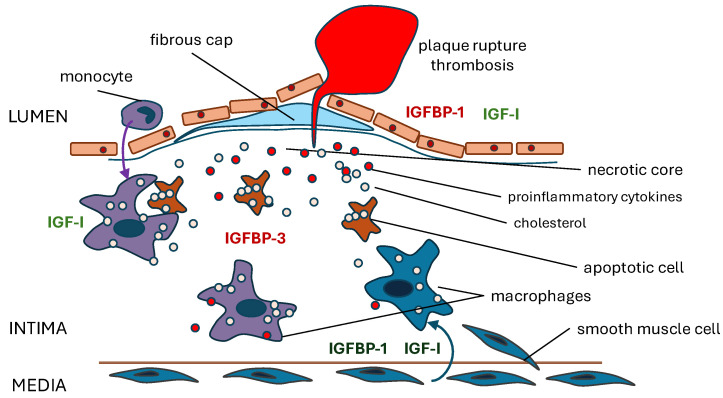
IGF system in the pathogenesis of atherosclerosis.

Most cardiovascular diseases are caused by atherosclerosis within the walls of large arteries. It is likely that this process is initiated by endothelial dysfunction, which mediates arterial wall shearing stresses and proinflammatory cell recruitment and invasion [116]. Adipose tissue also contributes to the proinflammatory environment, with dysregulated adipokine production in obesity playing a crucial role [117]. In addition to monocytes from the circulation, smooth muscle cells (VSMCs) are recruited from the arterial wall, and within the intima, both cell types make a phenotypic switch to express macrophage markers that can bind to modified low-density lipoproteins and that have highly proinflammatory characteristics [118]. In more advanced disease, vulnerable plaques, which are lesions consisting of a lipid core with active inflammation and a thin fibrous cap, are formed (illustrated schematically in Figure 2). Evidence suggests that overall, IGF-I has beneficial effects, reducing plaque burden and increasing plaque stability by a variety of mechanisms [3,119]. IGF-I reduces monocyte/macrophage recruitment, production of proinflammatory cytokines, conversion into lipid-laden foam cells and contribution to matrix degradation. IGF-I promotes SMC migration and proliferation and VSMC-dependent matrix deposition. These effects on VSMCs are of particular importance to plaque stability.

Recent research suggests that IGFBP-3 secretion by senescent cells in the fibrous cap antagonizes IGF-I-mediated switching of VSMCs from a contractile to a promigratory state, leading to speculation that inhibiting IGFBP-3 action might be of therapeutic benefit in atherosclerosis [120]. While IGFBP-1, produced locally or derived from the circulation, could, theoretically, act in a similar way, there is also evidence that IGFBP-1 may have IGF-independent effects on VSMCs. IGFBP-1 gene expression is increased in carotid plaques compared to normal arteries [98]. IGFBP-1 expression by VSMCs was stimulated in vitro by IL-1β, IL-6 and TNFα; furthermore, IGFBP-1 stimulates smooth muscle cell proliferation independently of the type 1 IGF receptor, an effect that was inhibited by an antibody to the α5β1 integrin receptor [98]. This would suggest a local protective role for IGFBP-1.

There is evidence that IGFBP-1 has direct effects on vascular endothelium. In isolated segments of mouse aorta from wild-type and IGF1R knockout mice, overexpression of human IGFBP-1 increases endothelial nitric oxide production independently of IGF-I and protects against atherosclerosis [121]. Expression of human IGFBP-1 in male mice improves vascular endothelial cell migration and proliferation [101]. Ligands of the Jagged families induce Notch signaling, which has an important role in endothelial and VSMC responses [122]. Jagged1 conditional knockdown in endothelial cells causes thickening of the vessel wall in mice [123]. Inhibition of Jagged1 leads to downregulation of IGFBP-1 mRNA and protein expression in human coronary arterial endothelial cells in culture, and IGFBP-1 promotes proliferation and protects cells against H_2_O_2_-induced senescence [66]. Exposure of cardiac microvascular endothelial cells to hypoxia/reoxygenation is an in vitro model of endothelial reperfusion injury [124]. IGFBP-1 and IL-6 secretion are upregulated under these conditions.

We speculate that IGFBP-1 may have different endocrine and paracrine roles in atherosclerosis. It is relevant that insulin enhances the passage of IGFBP-1 across the endothelium [125]. Further research is required in this area.

## 6. Concluding Comments

Cross-sectional and prospective studies demonstrate associations between IGFBP-1 and cardiovascular diseases. Inconsistencies in these relationships are likely explained by the changes in insulin sensitivity and the presence of chronic inflammation.

IGFBP-1 is a marker of hepatic insulin action. Under conditions of normal glucose tolerance and in the absence of inflammation, IGFBP-1 reflects whole-body insulin sensitivity. In the presence of peripheral insulin resistance, i.e., decreased responsiveness to insulin-mediated glucose disposal or adipose tissue lipolysis, insulin concentrations increase and IGFBP-1 is suppressed. IGFBP-1 is a better marker of insulin action than insulin itself. Fasting serum IGFBP-1 is more strongly correlated with insulin-mediated glucose disposal during a euglycemic insulin clamp than fasting insulin or HOMA [126]. Conversely, proinsulin has lower hepatic extraction and may have a stronger relationship with IGFBP-1 than insulin [44,50,51]. Proinsulin, and not IGFBP-1, is an independent predictor of all-cause mortality in a rural population of 66- to 81-year-olds [127].

In hepatic insulin resistance, the relationship between IGFBP-1 and insulin changes, with higher concentrations of insulin required to suppress IGFBP-1 secretion. In the presence of inflammation and increased production of proinflammatory cytokines, IGFBP-1 concentrations may also be disproportionately elevated compared to insulin. Awareness of these relationships between IGFBP-1 and insulin is important in considering the potential of IGFBP-1 as a biomarker of disease in population studies and in personalized medicine. Insulin resistance and chronic low-grade inflammation are key features of the metabolic syndrome. Hepatic fat accumulation, and, therefore, hepatic insulin resistance are also often present. Therefore, the interpretation of circulating IGFBP-1 concentrations depends on the extent of each of these phenomena.

Effective management of cardiometabolic diseases rests on prevention, and identifying predictive markers for those at risk is key. Low IGFBP-1 is likely to be a useful early marker of metabolic syndrome, and, therefore, disease risk. However, its role as a predictive marker will change as the features of the metabolic syndrome emerge. If hepatic insulin resistance and inflammation are dominant features, IGFBP-1 concentrations will be relatively high and may predict poor outcomes. In the presence of features of the metabolic syndrome, we recommend that IGFBP-1 be combined with other biomarkers and suggest that future research focuses on identifying the best combination. Other IGF system components might be considered in multimarker models; indeed, IGFBP-1 may be less useful. One study in a community setting has shown that, while IGFBP-1 alone predicted incident heart failure and cardiovascular disease mortality, IGFBP-2 and IGF-I were more useful in a multimarker model [57]. IGFBP-4, IGFBP-4 fragments and the IGFBP-4 protease PAPPA are also candidate markers [88,128]. The inclusion of IGF-II, which has distinct roles in metabolism [129], and other insulin-regulated hepatic proteins such as sex-hormone binding protein (SHBG) should be considered in these models. It has been shown that low SHBG might be a better predictor of cardiovascular mortality than IGFBP-1 [130]. The response of IGFBP-1 to food intake also limits its value in multimarker models and there are other potential cardiovascular biomarkers that are only modestly altered [131]. Thus, the evidence that IGFBP-1, alone, is a good marker of cardiovascular risk is weak; however, it is possible that it would be useful in multimarker models.

Biomarkers of cardiovascular disease should present an advantage over classical risk factors. We also recommend that future research should compare any models with those based primarily on clinical history and examination. It has been shown that a non-laboratory-based risk assessment that can be undertaken in a primary care consultation (age, sex, smoking, diabetes, blood pressure and family history of cardiovascular disease) was at least as good as a biochemistry panel that included IGFBP-1 or further sophisticated measurements, e.g., endothelial dysfunction [132]. The cost-effectiveness of any panel of predictive markers that are identified should be included in future research.

Consideration could be given to serial measurements of fasting IGFBP-1 as a monitoring biomarker [133]: more than one IGFBP-1 measurement, alone or with other markers, e.g., one year apart might help in assessing cardiometabolic health [76]. We propose that another approach to assessing cardiovascular risk would be to incorporate IGFBP-1 in dynamic studies of glucose tolerance and insulin sensitivity. Impaired glucose tolerance at one and two hours is associated with worse cardiometabolic risk, higher proinflammatory cytokines and lower IGF-I [134,135]. The combination of IGFBP-1 and ghrelin 2-h post-oral glucose tolerance test tends to predict cardiovascular outcomes better than fasting levels of these proteins [136]. It has previously been suggested that, in women with central adiposity, failure of IGFBP-1 to be suppressed by more than 40% after an oral glucose challenge identifies a group at high risk of increasing abdominal adiposity and diabetes [36]. An oral glucose tolerance test with measurement of glucose, proinsulin and IGFBP-1 might therefore identify those at increased cardiometabolic risk. The inclusion of one or more pro-inflammatory cytokines that are associated with the metabolic syndrome, e.g., CRP, and/or likely to increase IGFBP-1 production, e.g., IL-6, could help in assessment at the individual level. Non-invasive measurement of liver fat content might also be a useful addition.

Finally, IGFBP-1 levels are influenced by sex and age, and both also determine cardiometabolic risk, patterns of disease presentation and participation in research [54,55,56]. Particular attention should be paid to controlling for the influence of these factors in future studies of IGFBP-1 and cardiovascular diseases.

## 7. Conclusions 

There are associations between IGFBP-1 and cardiovascular diseases, however the evidence that IGFBP-1, alone, is a good biomarker is not strong. While lower circulating IGFBP-1 concentrations are associated with an unfavorable cardiometabolic risk profile, higher IGFBP-1 predicts worse cardiovascular disease outcomes. These changing relationships are explained by alterations in hepatic insulin sensitivity and the presence of chronic inflammation. Therefore, we recommend evaluation of dynamic approaches, such as simultaneous measurements of fasting IGFBP-1 and proinsulin level in response to an oral glucose challenge, as well multimarker approaches incorporating markers of inflammation.

## Data Availability

No new data were created or analyzed in this study. Data sharing is not applicable to this article.
